# Evolution of the Moral Lexicon

**DOI:** 10.1162/opmi_a_00164

**Published:** 2024-09-15

**Authors:** Aida Ramezani, Jennifer E. Stellar, Matthew Feinberg, Yang Xu

**Affiliations:** Department of Computer Science, University of Toronto, Toronto, Canada; Department of Psychology, University of Toronto, Toronto, Canada; Rotman School of Management, University of Toronto, Toronto, Canada; Cognitive Science Program, University of Toronto, Toronto, Canada

**Keywords:** language and morality, moral lexicon, moral foundations, lexical evolution, metaphor

## Abstract

Morality is central to social well-being and cognition, and moral lexicon is a key device for human communication of moral concepts and experiences. How was the moral lexicon formed? We explore this open question and hypothesize that words evolved to take on abstract moral meanings from concrete and grounded experiences. We test this hypothesis by analyzing semantic change and formation of over 800 words from the English Moral Foundations Dictionary and the Historical Thesaurus of English over the past hundreds of years. Across historical text corpora and dictionaries, we discover concrete-to-abstract shifts as words acquire moral meaning, in contrast with the broad observation that words become more concrete over time. Furthermore, we find that compound moral words tend to be derived from a concrete-to-abstract shift from their constituents, and this derivational property is more prominent in moral words compared to alternative compound words when word frequency is controlled for. We suggest that evolution of the moral lexicon depends on systematic metaphorical mappings from concrete domains to the moral domain. Our results provide large-scale evidence for the role of metaphor in shaping the historical development of the English moral lexicon.

## INTRODUCTION

Morality is a fundamental aspect of human society and cognition. From an evolutionary perspective, the emergence of morality helped early humans survive by allowing them to take advantage of the benefits of group living (Haidt, 2007), and create shared expectations of how to treat one another (Tomasello, 2016; Tomasello et al., 2012). As a result, humans have been able to exploit collective opportunities (e.g., big game hunting) and defend against collective threats (e.g., invasion by other groups). How exactly humans developed these complex moral systems is not well understood, but many believe that it stemmed, in part, from language. Through communication, language may have facilitated the emergence of shared systems of moral norms and helped uphold them through rewarding moral behavior and punishing immoral behavior (Li & Tomasello, 2021; Poulshock, 2006).

Despite the intimate connection between language and morality, how the moral lexicon—the words people use with moral connotations like *impure* or *murder*—has evolved over time is not clearly understood. Here, we investigate this question and study the historical evolution of words commonly used in English moral rhetoric, which form the building blocks of moral language. We propose that the evolution of moral words might critically depend on metaphorization, or our cognitive capacity to ground abstract moral thoughts in concrete experiences. Our theorizing builds on work that explores the relationship between morality and metaphor from Conceptual Metaphor Theory (Lakoff & Johnson, 1980). This theory holds that people interpret abstract domains through concrete and perceptual experiences, which is enabled by our ability to identify structural similarities between domains (Gentner, 1983). Recent work in social psychology has proposed similar views suggesting that moral concerns (e.g., purity) are associated with concrete and physical experiences (e.g., physical dirtiness) (Lee & Schwarz, 2010a, 2010b, 2021).

Empirical work has shed light on the cognitive role of metaphor in moral judgment, but to our knowledge, there has been no comprehensive study examining the role of metaphor in the evolution of the moral lexicon. Recent work has applied quantitative analyses to characterize the historical changes in different moral foundations based on the frequencies of words that instantiate these foundations (Wheeler et al., 2019). While such analyses provide a description for the historical trends of using the moral lexicon, it has assumed that the meanings of these words stay constant over time. Here, we critically assess this assumption by investigating whether and how (moral) words might have gained moral meaning. Specifically, we examine the role of metaphor in shaping the formation of the moral lexicon. We hypothesize that the modern moral lexicon has gained its moral meanings through metaphorical mappings of concrete experiences, i.e., concrete and grounded concepts used metaphorically to refer to abstract moral concepts. Our hypothesis is rooted in the view that meaning shift from concrete to abstract domains makes communication efficient since it is easy to achieve shared references based on concrete experiences (Thibodeau & Durgin, 2008). Therefore, metaphorization may offer one possible mechanism for the formation of the moral lexicon. This concrete-to-abstract meaning shift has been shown to be a primary force in the historical metaphorical mappings in English (Xu et al., 2017). Moreover, concrete concepts are also shown to emerge earlier compared to abstract concepts in language acquisition and formation of the mental lexicon (Ding et al., 2023; Fisher & Gleitman, 2002; Stella et al., 2018). Therefore, if words acquire moral meanings through metaphorization, we expect their historical trajectories to exhibit similar directionality.

Although work in historical linguistics suggests that metaphor is a key force underlying temporal changes in word meaning (Claudi & Heine, 1986; Geeraerts, 2006; Sweetser, 1990; Urban, 2014), some previous studies have shown that the English lexicon, on average, has gained concreteness (as opposed to becoming more abstract due to metaphorization) over time (Hills & Adelman, 2015; Snefjella et al., 2019). Therefore, it is unclear whether moral words tend to resist this overall trend toward concreteness and become more abstract.

**Figure 1.**
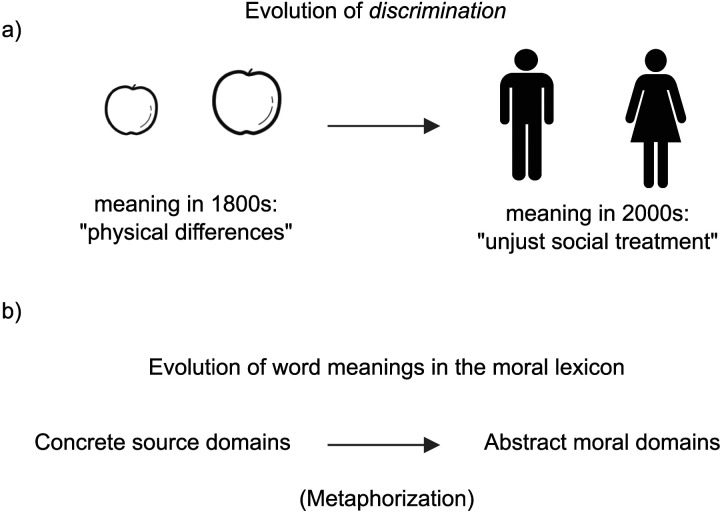
An illustration and overview of our main hypothesis. a) Illustration of moral meaning change in the English word *discrimination*. In the 1800s, *discrimination* was used to refer to making distinctions between things (e.g., fruits), but in the 2000s, it is commonly used to refer to unjust treatment of a person or a group. b) Overview of our theoretical hypothesis about the role of metaphorization in shaping the evolution of the moral lexicon.

Figure 1 illustrates our overall hypothesis and provides an example of metaphorical semantic change in the English moral word *discrimination*. While it originally expressed the concrete notion of disparity among physical objects 200 years ago, *discrimination* has since taken on the more abstract meaning of unjust social treatment, and is now a common subject in moral discussions, presumably because people metaphorically mapped its meaning from the physical (source) domain to the moral (target) domain.^1^

In all, our goal is to investigate the role of metaphor in moral semantic change and moral world formation, aiming to provide a more in-depth account of how metaphorical mappings have facilitated moral language development over time. Across four studies, we systematically investigate whether words take on abstract moral meanings from concrete grounded experiences through metaphorization. Study 1 constructs time courses of moral relevance and probes historical shifts from concrete to abstract meanings as words undergo moral semantic change. Building on this, Study 2 explores how moral word meanings shift from concrete to abstract semantic domains. Study 3 examines known cases of metaphorical mappings in the moral domain as attested in dictionaries, providing evidence for concrete-to-abstract shifts in the metaphorization of moral concepts. Finally, Study 4 explores the role of metaphor and concrete-to-abstract shifts in the formation of compound moral words. For these studies, we focus our analyses on the English moral lexicon, although we expect these findings to generalize across languages.

## MATERIALS

We draw on diverse datasets to test our hypothesis. We summarize these datasets with their sources, and specify how they relate to our case studies in Table 1.

**Table 1.** Summary of the datasets and their usages in this study.

Dataset	Usage, Study	Reference
Moral Foundations Dictionary (MFD)	Moral lexicon, Studies 1, 2, and 4	Graham et al. (2009), Frimer et al. (2019)
Historical Thesaurus of English (HTE)	Moral lexicon, Studies 1, 2, and 4	Kay et al. (2020)
Neutral word dataset	Neutral centroid, Study 1	Xie et al. (2019), Warriner et al. (2013)
Intercontinental Dictionary Series (IDS)	Semantic grouping, Study 2	Key and Comrie (2015)
Metaphor Map of English (MME)	Meptaphorical mappings between semantic domains, Study 3	Alexander et al. (2015)
LADEC: The Large Database of English Compounds (LADEC)	Compound analysis, Study 4	Gagné et al. (2019)
Concreteness ratings	Word concreteness, Studies 1, 2, and 4	Brysbaert et al. (2014)
Domain concreteness ratings	Semantic domain concreteness, Study 3	Xu et al. (2017)
Valence ratings	Neutral centroid and valence analysis, Study 1	Xu et al. (2017)

### Dictionaries

#### Moral Foundation Dictionary (MFD).

We use one of the largest lexical resources of contemporary moral terminologies in English, the Moral Foundations Dictionary (MFD), which was developed along with the Moral Foundations Theory (MFT) (Graham et al., 2013). Moral Foundations Theory proposes five conceptual foundations of morality that help explain similarities and differences in people’s moral views. These moral foundations, each comprised of virtues and vices, are Care/Harm, Fairness/Cheating, Authority/Subversion, Loyalty/Betrayal, and Sanctity/Degradation. In line with these moral foundations, the MFD provides a large vocabulary of English words thought to be commonly used to express moral concerns in linguistic communication (Graham et al., 2009). For example, words such as *compassion* and *brutality* represent virtues and vices in the Care/Harm foundation, while words like *duty* and *overthrowing* represent virtues and vices in the Authority/Subversion foundation. We use the most recent version of the MFD (MFD 2.0) as the basis of the modern English moral lexicon. We made this choice due to its broader coverage and greater empirical validity than the initial version (Frimer et al., 2019). While the MFD 1.0 includes around 32 words per moral foundation. The MFD 2.0 averages around 210 words per construct included specifically to capture prototypical moral concepts. This new version of MFD has also been shown to reflect crowd-sourced moral sentiments more accurately compared to the previous versions.

Large-scale analyses of morality have shown that computational models that use the MFD as their source of the moral lexicon can effectively make inferences about people’s moral concerns across various contexts (Garten et al., 2016; Hoover et al., 2020; Liscio et al., 2023; Mendelsohn et al., 2020; Mooijman et al., 2018; Ramezani et al., 2021; Rezapour et al., 2019). Other studies have further examined the fluctuation of MFD word frequencies with respect to each moral foundation over the twentieth century (Wheeler et al., 2019), and investigated temporal changes in moral sentiment of different concepts from their representations in historical textual corpora (Xie et al., 2019). However, these previous studies, assume that the meanings of moral words have remained unchanged over time, which we believe is not the case. In general, our focus differs from previous work in text-based studies of moral foundations because we are precisely interested in understanding how the moral lexicon itself develops as opposed to how linguistic communication reflects moral views.

#### Historical Thesaurus of English (HTE).

We consider a second large resource of the moral lexicon drawing words from the Historical Thesaurus of English (HTE) (Kay et al., 2020). The HTE offers a hierarchical conceptual taxonomy of more than 800,000 English words with their usages recorded over the past 1,300 years. The HTE initially groups words into three broad categories: “The World”, “The Mind”, and “Society”. These categories are then divided hierarchically from more general to more fine-grained ones. The semantic domain of “Morality” appears at the second level under the hierarchy within “Society”. From this category, we extracted around 6,000 words with usages recorded from Old English to the present day (e.g., *expiation*). We then removed archaic words which are out of the scope (of our corpus analysis), and excluded a set of 261 overlapping words with the MFD, which reduced the size of this moral lexicon to 1,722 words in total.

#### Neutral Word Dataset.

Following the methodology in Xie et al. (2019), we constructed a set of neutral words using a large-scale dataset of empirical valence ratings from Warriner et al. (2013). This dataset provides valence ratings for around 14,000 English words, from which we selected 1,200 words that fall within 0.1 standard deviations from the mean valence ratings. We contrast these neutral words with the moral lexicon in Study 1.

#### Intercontinental Dictionary Series (IDS).

This dataset is typically used for studying cross-linguistic semantic grouping over a large set (1,310) of concepts (e.g., *earth*, *worship*). IDS groups these concepts into 22 different semantic domains ranging from highly concrete such as ‘Animals’ and ‘The physical world’ to abstract such as ‘Law’ and ‘Religion and belief’ (Key & Comrie, 2015).^2^ We use IDS to complement our corpus-based analysis, and investigate concrete-to-abstract changes in moral words within different semantic domains in Study 2.

#### Metaphor Map of English (MME).

We use the Metaphor Map of English (MME) database (Alexander et al., 2015) to study historical metaphorical mappings of the moral domain in Study 3. The MME is one of the largest resources documenting more than 14,000 metaphorical mappings between 415 semantic domains from Old English to the present day recorded by lexicographers that have expertise in different periods of English. Each recorded metaphorical mapping in MME is annotated with a source domain and a target domain, along with the directionality of mapping (e.g., source → target), and example English words that were identified to exemplify that metaphorical semantic change (e.g., the word *unhuman* exemplifies the metaphorical mapping from ‘Humankind’ to ‘Moral Evil’).

#### Large Database of English Compounds (LADEC).

The LADEC dataset (Gagné et al., 2019) contains over 8,000 English compound words with attributes such as the degree of semantic transparency for the compounds and their constituents, and their frequencies in different text corpora. The semantic transparency variable estimates the degree to which the meaning of a compound word can be predicted from its constituents. The semantic transparency ratings in this dataset are collected from native English speakers, where human annotators are given a compound word and have to rate how predictable the meaning of the compound is from the meaning of its two constitutes. LADEC further provides frequencies for the compound words and their constituents from British National Corpus (BNC)^3^, as well as an American-English corpus collected from more than 1.10 billion English posts on Facebook (Herdağdelen & Marelli, 2017). We use LADEC to study moral word formation in Study 4.

### Ratings of Concreteness and Valence

#### Concreteness Ratings.

We use a database of concreteness ratings collected in Brysbaert et al. (2014) in Studies 1, 2, and 4. This dataset provides crowd-sourced concreteness ratings for 40,000 English words from native English speakers. Concreteness ratings in this dataset represent human judgments that range from 1 (very abstract concepts) to 5 (very concrete or tangible concepts).

#### Domain Concreteness Ratings.

We extract the human-annotated concreteness ratings of the semantic domains in MME from existing work (Xu et al., 2017) in Study 3. These concreteness ratings are crowd-sourced from native English speakers that range from 1 (highly abstract) to 7 (highly concrete).

#### Valence Ratings.

We use a large-scale dataset of empirical valence (i.e., degree of pleasantness) ratings in Study 1 from Warriner et al. (2013), which provides valence ratings for around 14,000 English words. These valence ratings range from 1 (highly negative valence) to 9 (highly positive valence concepts).

## METHODS

In this section, we provide an overview of the methodologies used in each of the four case studies. We make publicly available the code and data for replicating our analyses: https://osf.io/mnsjk/.

### Study 1

We use the MFD and HTE (see Materials section) as two sources of English moral lexica. These two databases differ from one another offering complementary tests of our hypotheses. The MFD provides us with a set of modern moral words gathered by moral psychologists, while the HTE enriches our analysis with historical and linguistic knowledge of the English moral lexicon. To quantify the meanings of these words from text through historical periods, we construct diachronic word embeddings from the HistWords project (Hamilton et al., 2016). Specifically, in each decade between 1800 and 1990, diachronic word embedding provides a latent high-dimensional representation of a word’s meaning from its usages (or contexts) in Google Books Ngram dataset (Michel et al., 2011), which allows us to track and compare semantic changes over historical times. In other words, at each decade, words can be represented within a semantic vector space where words closer in meaning have more similar representations compared to words with more disparate meanings.

To analyze moral semantic change, we use existing methods from natural language processing specifically diachronic word embeddings to construct moral time courses. Following the methodology in Xie et al. (2019), we classify words into two semantic groups at each historical time point: ‘moral’ and ‘neutral’ centroids. The ‘moral’ centroid is represented by the average word embedding of the 1,930 MFD words signifying modern moral concepts in our semantic vector space. To construct the ‘neutral’ centroid, we use neutral words (described in the Materials section) and compute the average word embedding of these words. This centroid represents modern neutral concepts that do not elicit strong positive or negative emotions and has been shown to effectively distinguish morally relevant from non-moral concepts (Xie et al., 2019).

For the two centroids to represent present-day moral and neutral concepts in the semantic vector space, following (Xie et al., 2019), we use word embeddings from the most contemporary decade (i.e., 1990s) in the HistWords project (Hamilton et al., 2016), and keep them fixed in our analyses. We then approximate words’ moral relevance in historic time points based on their proximity to the centroids. Formally:

$$\text{proximity}\left(c,w,t\right)=\text{exp}\left(-∥V_{t}\left(w\right)-E\left[S_{c}\right]∥\right)$$ (1)

$$p\left(c=\text{moral}|w,t\right)=\frac{\text{proximity}\left(c=\text{moral},w,t\right)}{\text{proximity}\left(c=\text{moral},w,t\right)+\text{proximity}\left(c=\text{neutral},w,t\right)}.$$ (2)

**Figure 2.**
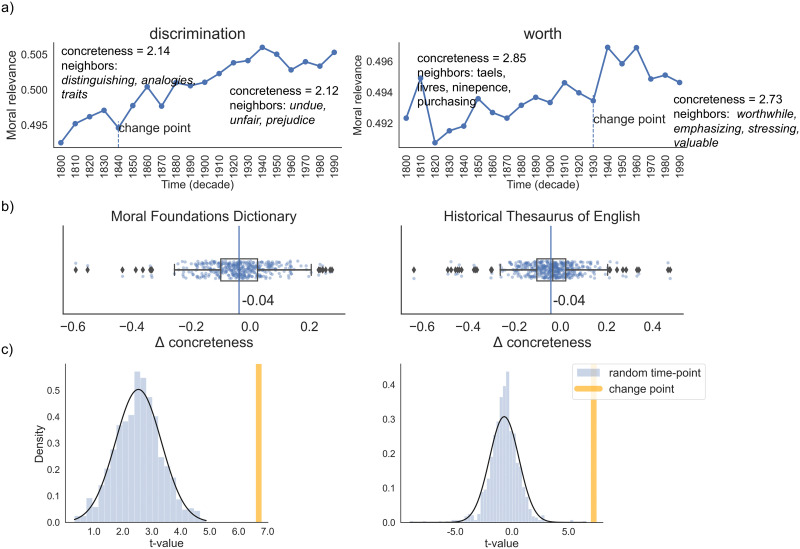
Summary of evidence for concrete-to-abstract shift in moral words. a) Illustration of concreteness shift in moral semantic change of two example English words *discrimination* and *worth*. Each panel shows the time course of moral relevance of a word’s meaning over the past two centuries. The vertical dash line marks the change point detected in the time course. b) Boxplots of concreteness change in the population of words that exhibit moral semantic change. Each dot shows the change in concreteness of a word’s semantic neighbors before and after the change point in its time course of moral relevance. The vertical line marks the mean indicating a statistical tendency of concrete-to-abstract shifts around the change points. c) Randomized test on concreteness change in the moral words. The yellow vertical bar shows the attested *t* value from a paired *t* test on concreteness differences before and after the change points in the moral time courses of words taken from the two resources. The histogram shows a null distribution of similar *t* values derived from using random time points instead of the actual change points.

Here, *V*_*t*_(*w*) is the word embedding for *w* (e.g., *discrimination*) at time *t* (e.g., 1890s) taken from the diachronic embeddings, and *E*[*S*_*c*_] is the average word embedding for the centroid *c*, where *c* is either ‘moral’ or ‘neutral’. As specified in this equation, we transform the Euclidean norm of the distance vector ∥*V*_*t*_(*w*) − *E*[*S*_*c*_]∥ to probabilities using a softmax function. We refer to *p*(*c* = *moral*|*w*, *t*) as a score of “moral relevance” since our model approximates the extent to which historical word meanings are relevant to modern moral concepts. To estimate moral relevance time courses of English words, we compute *p*(*c* = *moral*|*w*, *t*) incrementally for *t* in each decade in the 1800s–1990s. Figure 2a shows an example application of this framework for the words *discrimination* and *worth*.

To detect the time point (i.e., a decade) of moral semantic change, for each moral word, we apply an automatic change point detection algorithm (Kulkarni et al., 2015). This algorithm identifies statistically significant change points given a time series when the mean of the time series after the change point is different from the mean of the time series before the change point. We apply this change point detection algorithm to the moral relevance time courses of words, and refer to the most salient change point as the time point of moralization.

### Study 2

Complementary to our corpus-based analysis in Study 1, here, we consider a second dictionary-based study where we investigate concrete-to-abstract changes in moral words meanings by characterizing a wide range of semantic domains. For this purpose, we use IDS (see Materials section).

Similar to our methodology in Study 1, we create 22 centroids representing the semantic domains in IDS. To quantify moral words’ meaning change concerning these semantic domains, we measure their semantic proximity to each centroid before and after the detected moralization time point of that word. The semantic proximity of a word *w* to each centroid *c* at a time point *t* is defined as ‘domain proximity’, estimated formally as *p*(*c*|*w*, *t*) ∝ *exp*(−∥*V*_*t*_(*w*) − *E*[*S*_*c*_]∥) where *V*_*t*_(*w*) is the word embedding for *w* at time *t* using the diachronic embeddings, and *E*[*S*_*c*_] is the average word embedding of the centroid *c*. We define Δ*domain proximity* as the relative change in domain proximity before and after words’ moralization time points. A positive Δ*domain proximity* indicates that a word shifts meaning toward a domain after the change point, and a negative Δ*domain proximity* indicates meaning shift in the opposite direction by moving away from a domain. We also estimate the average concreteness of a semantic domain by taking the average of the concreteness ratings of its concepts using the dataset in Brysbaert et al. (2014) (see Materials section). These concreteness ratings are provided in Table 7 in the Supporting Information.

### Study 3

We use the MME database (Alexander et al., 2015) to study historical metaphorical mappings of the moral domain. We focus on the mappings that involve a sub-category of the ‘Morality’ semantic domain as either the source or the target. For example, the MME database identifies a metaphorical mapping from ‘Direction’ (source domain) to ‘Virtue’ (target domain), and lists the word *direct* as an exemplar for this metaphorization. Independently to the database, we extract the human-annotated concreteness ratings of the semantic domains in MME from existing work (Xu et al., 2017) (see Materials section).

### Study 4

We investigate the formation of the moral compound words and their level of abstraction compared to nonmoral compound words using the LADEC datase (Gagné et al., 2019).

## HYPOTHESIS EVALUATION AND RESULTS

### Study 1: Historical Evidence for Concrete-to-Abstract Meaning Change in Moral Words

To identify words that underwent moral semantic change during the period 1800s–1990s, we calculate the gross change in their moral relevance between the flanking decades 1800s and 1990s, using the formulation below, where *M*(*w*) represents the word’s *w* gross change in moral relevance, and *p*(*c* = *moral*|*w*, *t*) is calculated based on equation 2:

$$M\left(w\right)=\frac{p\left(c=\text{moral}|w,t=1990\right)-p\left(c=\text{moral}|w,t=1800\right)}{p\left(c=\text{moral}|w,t=1800\right)}.$$

Using bootstrapping and sampling, we identify words whose gross change *M*(*w*) were larger and lower than 2 standard deviations of the mean. We call these words ‘Changing’ and ‘Stable’ respectively. We use the ‘Changing’ words as the main focus of our analysis since they have faced significant increases in moral semantics during the 1800s–1990s. In addition, we discard all the words without a word embedding at any time point between 1800–1990, because it was impossible to conduct temporal analyses on these words. Overall we identify 396 ‘Changing’ words in MFD and 442 words in HTE. Similarly, we find 290 ‘Stable‘ words in MFD and 302 ‘Stable‘ words in HTE.

We examine the possibility that the distinction between ‘Stable’ and ‘Changing’ words is due to the fact that words in the former group were already associated with moral meanings at the initial time point, and thus, they did not further moralize over the period of 1800–1990. We observe that the moral relevance of the ‘Stable’ group is significantly higher than that of the ‘Changing’ group in 1800s (*t* = 14.34, *p* < 0.0001 for MFD; *t* = 16.27, *p* < 0.0001 for HTE), suggesting that the stable words may have already undergone moral semantic change by 1800s. Therefore, the association between their moralization and metaphorization cannot be analyzed in the time window we have.

We apply the change point detection algorithm described in the Methods section to the moral relevance courses of the words in the ‘Changing’ group. We then analyze the degree of concreteness change before and after words’ moralization change point and compare these paired values. Specifically, we take the average of the degree of concreteness three time points preceding and succeeding the change point. The concreteness of a word *w* at time point *t* is estimated by first finding its nearest semantic neighbors using the diachronic embeddings at *t* and then taking the average concreteness ratings among the neighboring words. We use an existing large-scale behavioral experiment (Brysbaert et al., 2014) for concreteness ratings. Following existing computational studies of semantic change (Xu et al., 2021; Xu & Kemp, 2015), we consider the word’s 100 nearest semantic neighbors for each word.

Using paired *t* tests on words’ concreteness reveals that moral words are significantly more concrete before the change points, and the degree of concreteness drops when they undergo moral semantic change (*t*(396) = 6.69, *p* < 0.0001 for MFD; *t*(442) = 7.22, *p* < 0.0001 for HTE). Figure 2a illustrates our analysis using two example words. In each case, we observe that concreteness drops in the semantic environment of a word as it acquires moral meanings after the historical change point was detected. For instance, the word *worth* neighbored words such as *taels*, *rubles*, and *livres* in 1900s, but neighbored words such as *valuable*, *stressing*, *treasure*, and even *dignity* by 1950s. We verify the robustness of our results by repeating the same analysis over different choices of neighborhood size. We also repeated this analysis for the ‘Stable’ word groups and found that the degrees of concreteness in these words tend to increase for all choices of moral lexicon and neighborhood size (see Supporting Information for details). These results provide support to the idea that modern English moral words originally had concrete meanings before they gained moral semantics. Figure 2b further shows the distributions of Δ*concreteness* for the word populations in MFD and HTE centering around negative values, which indicates an overall trend for concreteness to drop around the change point. Moreover, we find that the observed concrete-to-abstract shifts remain robust for different part-of-speech tags (verbs, nouns, and adjectives) in both MFD and HTE (see Supporting Information and Table 3 for details and results of this analysis).

To assess whether the concrete-to-abstract changes were specifically tied to the change points, we perform a randomized test on the degree of concreteness change. We repeat the *t* test analysis from the previous experiment at random time points (instead of the change points) for 1,000 trials. We use these randomized statistics (i.e., *t* values) to construct a null distribution for concreteness change. Figure 2c compares the null distribution from the random trials with the attested *t* value obtained by anchoring at the change point. In both MFD and HTE, our results show that the concreteness change at the point of change, denoted by the *t* values obtained initially (*t*(396) = 6.69 for MFD; *t*(442) = 7.22 for HTE), is significantly greater than random time points (*Z* = 5.19, *p* < 0.0001 for MFD; *Z* = 6.64, *p* < 0.0001 for HTE). This analysis confirms that there is a significant difference between the concrete-to-abstract change at the moral change point versus at random time points. The results further support that moral semantic change follows the concrete-to-abstract pattern, (rather than abstract-to-abstract), implying that moral semantics are grounded in concrete experiences. We also examined the role of valence in moral semantic change as an alternative to concreteness. Our results, details of which are outlined in Supporting Information, show that it is concreteness, rather than valence, that most robustly underlies the evolution of moral semantics.

### Study 2: Cross-Domain Word Meaning Change in the Moral Foundations Dictionary

To better understand how the concrete-to-abstract changes described relate to the process of metaphorization, we study how word meanings have shifted among different semantic domains in IDS. If metaphor underlies the concrete-to-abstract changes in moral words, we should expect systematic shifts in word meanings to take place from semantic domains that relate to concrete experiences toward those domains that are more abstract and morally relevant.

**Figure 3.**
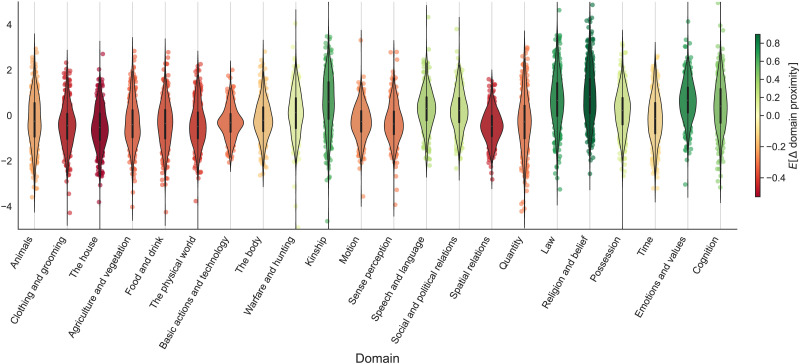
Visualization of historical cross-domain meaning changes in words from the Moral Foundations Dictionary. Color gradient indicates the change in semantic proximity of a word’s meaning to a domain (i.e., a dot on the graph). Red (or a negative value) indicates meaning shift away from a domain, and green (or a positive value) indicates meaning shift toward a domain. The horizontal axis orders the domains from the more concrete ones on the left to the more abstract ones on the right.

To quantify moral meaning change concerning these semantic domains, we measure the semantic proximity of a word to IDS domains before and after the detected change point of that word in its moral time course (see Methods section). Figure 3 summarizes the moral meaning change in MFD words for the 22 semantic domains in IDS. Overall, we observe the systematic patterns that 1) meanings of the moral words were more proximal to the concrete semantic domains before the change points, and 2) meanings of the moral words became closer to the abstract semantic domains after the change points. These patterns reflect the signatures of metaphorization where words derive their contemporary (abstract) moral meanings from concrete domains. For example, the MFD words tend to become closer to concepts under the domain ‘Religion and belief’ and ‘Law’, which are abstract domains, while moving away from concepts under the domains ‘Spatial relations’ and ‘Food and drink’, which are concrete domains. We also find that the MFD words tend to get closer to the semantic domain ‘Kinship’, which lies in the middle of the concrete-abstract spectrum. This could imply that when words gain moral semantics they are more regularly associated with concepts for social relations including family members or relatives. Correlating the concreteness ratings of the semantic domains with their average Δ*domain proximity* (i.e., the color legend in Figure 3) further shows that these variables are closely connected (Pearson’s *r* = −0.65, *p* < 0.01, *n* = 22). Namely, moral words become close to abstract semantic domains and further from the concreteness ones at the time points of moralization. This set of results provides further evidence for the role of concrete-to-abstract changes in the evolution of the moral lexicon: when a word undergoes moral semantic change, it tends to extend its original meaning from concrete domains toward more abstract domains.

### Study 3: Asymmetries in the Historical Metaphorical Mappings of the Moral Domain

Our results so far were informed by the analyses of historical text corpora that span the period 1800–1990. We now extend our period of investigation to the past millennium by examining a database that records metaphorical mappings of the moral domain through the historical development of English.

We query the Metaphor Map of English (MME) database, which is a dictionary-based resource of metaphorical mappings (see Materials section). For our analysis, we consider all 273 recorded cases of metaphorical mapping that include the domain of ‘Morality’ as either the target domain or the source domain. In addition, we take the human ratings of concreteness for all the domains concerning these recorded cases from existing work on MME (Xu et al., 2017).

To evaluate our hypothesis, we focus on examining the asymmetries in the metaphorical mappings of morality in two respects: 1) asymmetry in the directionality of metaphorical mapping, namely whether domains of morality predominantly serve as the target domain as opposed to the source domain in the recorded cases of metaphorical mapping; 2) asymmetry in concreteness, namely whether in cases of metaphor where morality is the target domain, the source domain tends to be more concrete than the target domain.

**Figure 4.**
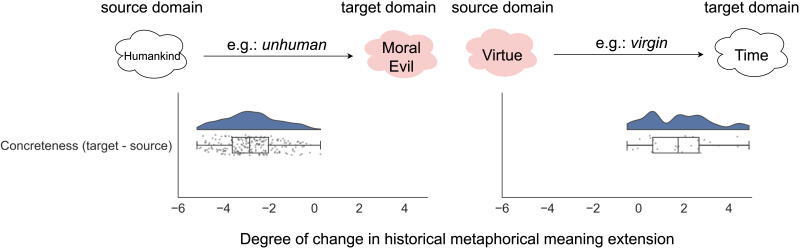
Evidence for asymmetry in the historical metaphorical mappings of the moral domain, with evidence that concrete → moral is the most prominent direction. The left panel shows that in a metaphorical mapping with morality as the target domain (e.g., ‘Moral Evil’), the source domain tends to be more concrete (e.g., ‘Human’). The right panel shows that in a metaphorical mapping with morality as the source domain (e.g., ‘Virtue’), the target domain tends to be more concrete (e.g., ‘Time’). The number of metaphorical mappings in this case is substantially lower than the case where morality is the target domain.

Figure 4 summarizes the results. Regarding asymmetry in directionality, we find that 242 out of 273 cases recorded the metaphorical mapping direction to be *X* → moral domain, where *X* is a non-moral source domain, and only 31 cases in the opposite direction where a moral domain serves as the source. This result shows a significant asymmetry (binomial test *p* < 0.0001, *n* = 273) in the directionality of metaphorical mappings into the moral domain, which confirms that morality is predominantly the target domain in the historical process of metaphorization for English moral words. Regarding asymmetry in concreteness, we find that when morality is the target domain (i.e., a word gains a morally relevant meaning through metaphor), the degree of concreteness is significantly higher in the non-moral source domains than in the moral target domains (*t*(242) = −33.37, *p* < 0.0001), meaning that words from concrete domains were used metaphorically in moral domains. We also find that when morality is the source domain, the target domain is significantly more concrete (*t*(31) = 6.54, *p* < 0.0001), suggesting that it is possible for moral words to extend toward concrete meanings (e.g., the word *virgin* has undergone a metaphorical semantic change from the ‘Virtue’ domain to ‘Time’ in usages such as ‘virgin land’). This set of results provides direct evidence for the role of metaphor in the evolution of moral word meanings in the English lexicon. In particular, we find that the concrete → moral is the predominant direction in metaphorical mappings involving moral concepts.

### Study 4: Evidence for Metaphorization in the Formation of Compound Moral Words

In addition to our analyses on the evolution of moral semantics, we assess whether the derivation of moral word forms might also be shaped by metaphor. Focusing specifically on compound words, we expect that if metaphor plays a role in moral word formation, these (abstract) moral words should be semantically related to their individual constituents drawing on more concrete ideas. Some examples of compound moral words include *blackmail* (MFD), *freeloader* (MFD), *whitewash* (HTE), *birthright* (HTE), etc.

To test our proposal, we use the LADEC dataset of English Compounds (Gagné et al., 2019), and analyze the degree of concreteness in the constituents of moral words. Specifically, we compare the human concreteness ratings of compound moral words to the average concreteness ratings of their constituents. Similar to Studies 1 and 2, the concreteness ratings are collected from a large-scale dataset of empirical concreteness ratings for the English words (Brysbaert et al., 2014) (see Materials section). Formally, if *w* is a moral compound with constituents *c*_1_ and *c*_2_, we define a metric called “degree of abstraction” for a word *w* as

$$\text{degree}\text{\_}\text{abstraction}\left(w\right)=\frac{\text{concreteness}\left(c_{1}\right)+\text{concreteness}\left(c_{2}\right)}{2}-\text{concreteness}\left(w\right).$$

A positive degree of abstraction means that the compound word as a whole is perceived more abstract (less concrete) than its constituents, while a negative degree of abstraction means that the compound word is less abstract (more concrete).

To enable a controlled analysis, we create two sets of baseline word groups by taking into account word frequency which correlates with (word) concreteness. We use the word frequencies in the British National Corpus (referred to as BNC frequency) and the American corpus of Facebook posts (Herdağdelen & Marelli, 2017) (referred to as American frequency) as provided in the LADEC dataset. To construct the first control group, we pair and compare each compound moral word with a compound non-moral word that is closest in word frequency (e.g., moral compound “safeguard”, BNC frequency = 650 vs. non-moral compound “wheelchair”, BNC frequency = 641). To construct the second control group, we pair and compare each compound moral word with a compound non-moral word that has the closest average frequency based on the two constituent words (e.g., moral compound “overthrow”, average constituent BNC frequency = 67,861 vs. non-moral compound “override”, average constituent BNC frequency = 67,818). Note that the frequencies of constituents are only available in the British National Corpus, while the frequencies of compound words are available in both the British National Corpus and the American corpus.

We use paired *t* tests to compare the degree of abstraction between compound moral words and control words. Results summarized in Table 2 show that the degree of abstraction in compound moral words (from MFD and HTE) is generally more than that in the two control groups. This finding suggests that moral words are statistically more reliant on concrete-to-abstract derivation in word formation from their individual constituents compared to non-moral counterparts in the lexicon. For instance, moral compounds such as “blackmail” are notably more abstract with respect to their constituents (i.e., “black” and “mail”, both concrete), compared to non-moral compounds such as “armchair” with respect to their constituents (i.e., “arm” and “chair”).

**Table 2.** Paired *t* test results from comparing the degree of abstraction in the formation of compound moral words and non-moral control groups. The variables *t* and *n* show the t-statistics and the number of word pairs for which the frequencies are available. Asterisks show the significance level after Bonferroni *p* value correction with the error rate *α* = 0.05.

Frequency source	Control group	Degree of abstraction
American corpus	Group 1	*t* = 2.90** (*n* = 45)
BNC	Group 1	*t* = 2.16 (*p* = 0.06, *n* = 32)
BNC	Group 2	*t* = 2.40* (*n* = 72)

We also examine the difference in the average concreteness ratings of the constituents between moral and control word groups. The idea is to sanity-check whether the constituents in these two groups are about the same in concreteness. Using paired *t* test, we find no statistical difference in the average concreteness ratings of the moral compounds and the frequency-controlled non-moral compounds (*t*(45) = −1.37, *p* = 0.176 using American corpus frequency; *t*(32) = −1.31, *p* = 0.199 using the BNC frequency).

In addition to abstraction gain, we assess semantic transparency of compound words to investigate the extent to which the meaning of a compound moral word can be construed successfully from the meaning of its two constituents (Gagné et al., 2019). For example, the meaning of “wrongdoer” is easily constructed from its constituents (“wrong” + “doer”) while the meaning of “honeymoon” seems unrelated to the meanings of “honey” and “moon”. The LADEC project reports this measurement on a scale of 0–100 where higher numbers represent higher semantic transparency with data from human annotators. If metaphor influences compound formation in the moral lexicon, we expect these words to draw on concrete constituent concepts but nevertheless deliver transparent moral meanings as a result. We find that compound moral words are just as semantically transparent as the non-moral control words (permutation test *p* = 0.79, repetition = 10,000). This means that the extent to which we can comprehend or infer the meaning of compound words from their constituents is similar for compound moral words and non-moral words.

Our findings provide evidence for the role of concrete-to-abstract metaphorical mappings in the formation of moral words: statistically speaking, the compound moral words gain abstraction from their (concrete) constituents, substantially more so than the compound non-moral words, through metaphorization. These observations suggest that metaphor is an effective cognitive device in structuring the meaning of compositional moral words (similar to how it facilitates meaning shift of moral words).

## DISCUSSION

Previous work demonstrated that there is an increasing tendency toward using concrete words over abstract words in English (Hills & Adelman, 2015; Snefjella et al., 2019) and that word meanings typically move from abstract to concrete over time (Snefjella et al., 2019). However, when it comes to moral words, we found the opposite pattern suggesting that our findings cannot be explained by the general trend in the evolution of the English language. The present research goes beyond past work which studied the rise of and fall of moral foundational lexicon over the past century by assuming moral word meanings stay constant (Wheeler et al., 2019). Instead, we investigate how words in the MFD and HTE have changed meaning over time. Our findings resonate with work on semantic change suggesting metaphor as a key mechanism in historical meaning change (Sweetser, 1990), whereby semantic changes in the moral domain can be a manifestation of that general cognitive process.

Generally, our results provide evidence for the significant role of metaphorical mappings through grounding moral meanings in concrete experiences. We find that the observed patterns throughout moral semantic change, are associated with metaphorization more than other semantic change alternatives. For example, semantic restriction (i.e., words’ meaning getting less generalized) and semantic extension (i.e., words’ meaning getting more generalized) cannot explain the observed concrete-to-abstract shifts. Another type of semantic change, that applies to morality, is semantic pejoration. During semantic pejoration, the sense of a word takes on more negative properties. As the result of our valence analysis implies, this can be a case for negative moral words. However, the concrete-to-abstract phenomenon is still captured in these words, suggesting the evolution of metaphorical mapping and semantic pejoration at the same time. We did not find any substantial evidence for word meanings gaining positive valence during moralization, therefore, semantic amelioration (i.e., words getting positive evaluations during the semantic change) is unlikely to explain the general trend of moral semantic change (see Supporting Information for details).

Regardless, we acknowledge that the patterns identified reflect strong statistical tendencies but not rigid laws, and propose a general association between moralization and metaphorization which has been possible through grounding moral meanings of words in their old concrete meanings. The exact causal relationships among these entities may be quite complex and potentially bidirectional. For example, although we found that moral word meanings transfer from the concrete to the moral domain far more often than the reverse, we did find cases where moral word meanings transferred from the moral domains to the concrete ones (Figure 4). When and why this reverse effect takes place can be a potentional direction for future work. Moreover, we do not consider cases where some historical moral words lose their usage in modern moral rhetoric. Future work can investigate the relationships that might predict when words gain or lose moral meanings.

In this work, we use the contemporary concreteness ratings of the words’ neighbors to determine the degree of concreteness, which assumes that the neighboring words themselves have not faced concreteness change during the time of investigation. Moreover, the change points we focused on are not the only historical times that words undergo semantic change. A word might moralize at some point in history yet experience other kinds of semantic change at different times. For instance, we did observe a tendency for concreteness to rise in later decades after the point of moralization, which aligns with existing studies suggesting a general increase in concreteness in the English lexicon. This concreteness change may be due partly to the fact that some moral words become more frequently used and applied to describe everyday scenarios.

In our study, we use moral lexica that are gathered by domain experts. However, previous work has critiqued the validity of dictionary-based approaches in moral inference due to the contextual nature of linguistic communication and the lack of intuitive human evaluation in developing these lexica (Garten et al., 2018; Hopp et al., 2021; Hopp & Weber, 2021). Moreover, our dictionary-based approach distinguishes our work from psychological studies of moralization that investigate how different concepts (e.g., smoking, vegetarianism) gain moral properties and become associated with people’s moral views (Feinberg et al., 2019; Rhee et al., 2019; Rozin, 1999). Future work can extend this line of research to investigate mechanisms that underline moralization of different concepts.

The observed shifts in concreteness and valence change remain robust across different parts of speech. While we relied on historical records for part-of-speech tags of HTE words, we derived these tags based on their modern usages for words in the MFD. Moreover, to isolate the effect of a word’s part of speech on its concreteness change, we excluded those with multiple recorded parts of speech. However, it is possible that as words gain or lose moral meanings over time, the frequency with which their different forms are used also changes, making their part of speech fluid. Exploring how words’ parts of speech evolve alongside their moral semantic changes through metaphorization could be a potential future extension of our work.

In sum, our work connects and extends two existing programs of research. Firstly, it connects to work examining the relationship between language and morality (Li & Tomasello, 2021; Poulshock, 2006), which points to language as an impetus for moral development. Secondly, it ties to concepts of grounding in morality (Lakoff, 1996; Lee & Schwarz, 2010a, 2010b, 2021), which proposes that metaphor influences moral judgment and behavior. While language may have helped morality evolve, our work suggests that cognitive processes such as metaphorization may have helped the language for morality evolve.

## CONCLUSION

Prior research shows that the historical frequencies of the moral lexicon fluctuate over time without specifying how the moral lexicon might have formed in the first place (Wheeler et al., 2019). Our work shows that moral word meanings have evolved through history. Our analyses of two large resources of moral vocabulary reveal that moral words tend to originate from concrete meanings and provides evidence for the role of metaphor in moral semantic change. Our analyses of compound moral words further suggest that metaphor plays a role in shaping compositional word derivation in the evolution of the moral lexicon. Future work can assess the generality of our findings to languages other than English and explore questions such as why certain words became moralized over time (e.g., *discrimination*), whereas others did not (e.g., *difference*). Such lexicalization strategies may be similar or different across languages, opening up new avenues for studying the evolution of language and morality across cultures.

## ACKNOWLEDGMENTS

We thanks Spike W. S. Lee for his helpful feedback on our manuscript.

## FUNDING INFORMATION

This work was supported by a NSERC Discovery Grant RGPIN-2018-05872, a SSHRC Insight Grant 435190272, and an Ontario ERA Award #ER19-15-050 to YX.

## AUTHOR CONTRIBUTIONS

A.R.: Conceptualization; Data curation; Formal analysis; Methodology; Writing – original draft. J.E.S.: Conceptualization; Writing – review & editing. M.F.: Conceptualization; Writing – review & editing. Y.X.: Conceptualization; Data curation; Formal analysis; Funding acquisition; Methodology; Writing - review & editing.

## DATA AVAILABILITY STATEMENT

Data and code for replicating our analyses are available at https://osf.io/mnsjk/.

## Notes

^1^ Some of the first records of using *discrimination* to refer to unjust racial treatments are found in the 1860s.^2^ https://ids.clld.org.^3^ https://www.natcorp.ox.ac.uk.

## Supplementary Material


