# Supplementary material for: Evolution of the Moral Lexicon
Source: Open Mind (Camb). 2024 Sep 15;8:1153–69. doi: 10.1162/opmi_a_00164 (PMC11441783; doi:10.1162/opmi_a_00164)
Supplement: Supplementary file 1 [file opmi-08-1153-s001.pdf]

Evolution of the moral lexicon

Supportive Information

Aida Ramezani[1*], Jennifer E. Stellar[2], Matthew Feinberg[3], and Yang Xu[1,4]

[1]Department of Computer Science, University of Toronto, Canada

[2]Department of Psychology, University of Toronto, Canada

[3]Rotman School of Management, University of Toronto, Canada

[4]Cognitive Science Program, University of Toronto, Canada

[*]Corresponding author email: armzn@cs.toronto.edu

In addition to our experiments on concreteness, here we examine valence (i.e., degree of pleasantness to capture the sentiment of a word) as a secondary factor to concreteness in metaphorical mapping. Our investigation on the role of valence in moral evolution is motivated by the role of semantic orientation in meaning change. Specifically, words can change meanings by gaining positive semantics (i.e., amelioration) or negative semantics (i.e., pejoration) (Cook and Stevenson, 2010). Valence has also been shown to play a role in metaphorical semantic over the past millennium as new senses become more emotionally engaging than the prior ones (Xu et al., 2017). Moreover, the literature of Concept Creep in the domain of psychology suggest that concepts related to Care/Harm (e.g., safety, bullying, trauma) tend to creep (or expand) their meanings toward new and less severe cases (Haslam, 2016; Haslam et al., 2020). Therefore, we can expect to observe changes in words' valence as they gain new moral meanings. However, it remains unclear whether these valence changes are positive or negative, and whether concreteness or valence plays a more predominant role in shaping the moral lexicon.

Similar to our analysis on concreteness change, we measure the valence change of a word by considering the average valence ratings of its 100 semantic neighbors before and after the change point. For this analysis, we also partition the words into morally positive and negative groups based on the available valence ratings (for HTE words) or the MFD categorization (for MFD words). Words from the moral foundations of Care, Fairness, Authority, Loyalty, and Sanctity are virtuous, and words from foundations of Harm, Cheating, Subversion, Betrayal and Degradation are vice. If a word was assigned to more than one moral foundation, we removed it from this experiment. The moral foundation information is not available for HTE words, so to determine moral polarity for these words, we use their valence

ratings (Warriner et al., 2013). If a word's valence rating is above 5 it is regarded as a positive word, and a negative word otherwise (the mean of valence ratings in the original dataset is 5.06, and the median is 5.2). We find that valence and concreteness are independent dimensions, confirmed in the insignificant results from χ^2 tests of independence between human ratings of concreteness and valence ($\chi^2 = 0.11$, $P = 0.74$ for MFD; $\chi^2 = 2.72$, $P = 0.10$ for HTE), where we take both kinds of ratings from existing large-scale behavioral experiments (Brysbaert et al., 2014; Warriner et al., 2013).

We first compare morally stable and changing word groups along three dimensions: moral relevance, concreteness and valence of semantic neighbors. We focus on analyzing the differences between these two word groups at the initial time point of our investigation, the 1800s. In particular, we examine the possibility that the distinction between moral stable and changing groups is due to the fact that words in the former group were already associated with moral meaning at the initial time point, and hence they did not further moralize over the period of 1800s-1990s. Overall, we observed that the moral relevance of the stable group is significantly higher than that of the changing group ($t = 14.34$, $p < 0.001$ for MFD; $t = 16.27$, $P < 0.0001$ for HTE). This confirms our expectation that the stable group became morally relevant earlier than the changing group. Similarly, we also observed that the stable group is significantly less concrete ($t = -4.27$, $p < 0.0001$ for MFD; $t = -5.80$, $p < 0.001$ for HTE), and less valenced ($t = -6.53$, $p < 0.0001$ for MFD; $t = -8.52$, $p < 0.0001$ for HTE) than the changing group. These results suggest that the stable words have already undergone the moralization process by the initial time point of our analyses. Since we focused on characterizing the process of moralization, we use the changing words for our analyses in the main text.

We evaluate the robustness of our findings on concreteness and valence changes during the process of moral meaning change. The analysis described in the main text was based on $k = 100$ nearest semantic neighbors for the changing word group. Here we repeated this analysis on both the changing words and the stable words for a range of semantic neighborhood sizes: $k = 25, 50, 100, 150$, or 200.

Tables II and III summarize the results for concreteness change and valence change respectively. We find that the degrees of concreteness drops significantly in the changing word group and robustly across the range of k values, while they remain stable (or show insignificant changes) or increase in most cases, in the stable word group. These results suggest that the attested drop in concreteness is only prominent and a product of the process of moral meaning change.

Additionally, Table IV summarizes the results from the t-tests when grouping words to virtuous and vicious categories with $k = 100$ nearest neighbors. We observe significant changes in both concreteness and valence for the morally negative words (e.g., *spoiled* shows a decrease in both concreteness and valence), but only significant changes in concreteness for the morally positive words (e.g., *impartial* shows a decrease in concreteness but not in valence). The valence changes in positive moral words are not statistically significant for both MFD and HTE words, meaning that while low valence words (i.e.,

Table I: Degree of concreteness and valence change as measured by paired t-tests for different part-of-speech tags. The variables t and n show the t-statistics and the number of samples for each test. Asterisks show the significance level after Bonferroni p-value correction with the error rate $\alpha = 0.05$, and 'n.s.' shows non-significant results.

Source of moral words	Variable	Verbs	Nouns	Adjectives	Adverbs
MFD	Concreteness	$t = 3.34^{**}$ ($n = 22$)	$t = 4.05^{***}$ ($n = 130$)	$t = 2.13^{\cdot}$ ($n = 60$)	($n = 3$)
	Valence	$t = 1.045$ (n.s.)	$t = 2.19^{\cdot}$	$t = 1.76$ (n.s.)	
HTE	Concreteness	$t = 2.46^{*}$ ($n = 65$)	$t = 5.12^{***}$ ($n = 163$)	$t = 3.79^{***}$ ($n = 111$)	$t = 1.94$ ($n = 22$)
	Valence	$t = 1.74$ (n.s.)	$t = 1.23$ (n.s.)	$t = 0.11$ (n.s.)	$t = 0.86$ (n.s.)

negative words) lose valence after their moralization time point, high valence words (i.e., positive words) do not undergo significant valence changes. These results indicate that concreteness change is central to the overall evolution of moral semantics, whereas valence change is mostly relevant to the negative change.

Finally, we grouped words in the MFD and HTE into different parts of speech (i.e., verbs, nouns, adjectives, and adverbs). For words in the MFD, we extracted their part-of-speech (POS) information from the WordNet dataset (Fellbaum, 2005) using the `NLTK` package. The POS information for the historical usages of words in the HTE was already available from Kay et al. (2020). We identified 93 words in the MFD and 85 words in the HTE with multiple parts of speech (e.g., *subordinate* and *defile*). These words were removed from the analysis to isolate the effect of different POS tags on concreteness and valence change. Table I provides the results of our concreteness and valence experiments for different word groups. As shown, the observed concrete-to-abstract shifts remain robust for verbs, nouns, and adjectives, although the adjective group in the MFD is only marginally significant after p-value correction ($p = 0.07$). There were also fewer adverbs in the MFD ($n = 3$) and HTE ($n = 22$) compared to other categories, and their concrete-to-abstract shifts are not statistically significant. While the nouns in the MFD show marginally significant drops in valence ($p = 0.06$), none of the other word categories exhibit valence change.

Table II: Degree of concreteness change as measured by paired t-tests for $k \in [25, 50, 100, 150, 200]$ nearest semantic neighbors. Asterisks show the significance level after Bonferroni p-value correction with the error rate $\alpha = 0.05$, and 'n.s.' shows non-significant results.

Source of moral words	Word group	$k = 25$	$k = 50$	$k = 100$	$k = 150$	$k = 200$
MFD	Stable ($n = 290$)	$t = -0.60$ (n.s.)	$t = -1.74$ (n.s.)	$t = -1.54$ (n.s.)	$t = -1.97$ (n.s.)	$t = -2.32$ (n.s.)
	Changing ($n = 396$)	$t = 3.27^{**}$ (n.s.)	$t = 5.72^{***}$	$t = 6.69^{***}$	$t = 6.49^{***}$	$t = 7.82^{***}$
HTE	Stable ($n = 302$)	$t = -2.75$ (n.s.)	$t = -1.96$ (n.s.)	$t = -1.78$ (n.s.)	$t = -1.70$ (n.s.)	$t = -1.83$ (n.s.)
	Changing ($n = 442$)	$t = 3.30^{**}$	$t = 5.74^{***}$	$t = 7.22^{***}$	$t = 8.42^{***}$	$t = 8.97^{***}$

Table III: Degree of valence change as measured by paired t-tests for $k \in [25, 50, 100, 150, 200]$ nearest semantic neighbors. Asterisks show the significance level after Bonferroni p-value correction with the error rate $\alpha = 0.05$, and 'n.s.' shows non-significant results.

Source of moral words	Word group	$k = 25$	$k = 50$	$k = 100$	$k = 150$	$k = 200$
MFD	Stable ($n = 290$)	$t = -1.25$ (n.s.)	$t = -1.77$ (n.s.)	$t = -1.37$ (n.s.)	$t = -1.13$ (n.s.)	$t = -1.22$ (n.s.)
	Changing ($n = 396$)	$t = 3.38^{**}$	$t = 4.90^{***}$	$t = 4.26^{***}$	$t = 4.89^{***}$	$t = 5.23^{***}$
HTE	Stable ($n = 302$)	$t = -1.06$ (n.s.)	$t = -2.27$ (n.s.)	$t = -2.97$ (n.s.)	$t = -2.56$ (n.s.)	$t = -2.32$ (n.s.)
	Changing ($n = 442$)	$t = 0.61$ (n.s.)	$t = 1.44$ (n.s.)	$t = 1.44$ (n.s.)	$t = 1.81$ (n.s.)	$t = 1.94$ (n.s.)

Table IV: Comparative statistics of historical changes in concreteness versus valence of moral words. Asterisks show the significance level after Bonferroni p-value correction with the error rate $\alpha = 0.05$, and 'n.s.' shows non-significant results.

Source of moral words	Moral polarity	$\Delta Concreteness$	$\Delta Valence$
Moral Foundations Dictionary	Virtue ($n = 254$)	$t = 4.56^{***}$	$t = -0.81$ (n.s.)
	Vice ($n = 142$)	$t = 5.27^{***}$	$t = 7.82^{***}$
Historical Thesaurus of English	Virtue ($n = 195$)	$t = 4.97^{***}$	$t = -1.40$ (n.s.)
	Vice ($n = 128$)	$t = 2.81^{*}$	$t = 2.12^{\cdot}$

Table V: Number of words with available concreteness ratings and their average concreteness ratings in each IDS domain.

Semantic domain	Word count	Average concreteness rating
The physical world	79	4.47
Kinship	67	4.034
Animals	97	4.798
The body	162	4.288
Food and drink	87	4.476
Clothing and grooming	66	4.643
The house	48	4.614
Agriculture and vegetation	69	4.574
Basic actions and technology	76	4.289
Motion	89	4.025
Possession	59	3.081
Spatial relations	88	3.461
Quantity	43	3.237
Time	64	3.026
Sense perception	55	3.612
Emotions and values	65	2.47
Cognition	61	2.134
Speech and language	48	3.545
Social and political relations	49	3.487
Warfare and hunting	44	4.051
Law	27	3.156
Religion and belief	30	3.129

References

Brysbaert, M., Warriner, A. B., and Kuperman, V. (2014). Concreteness ratings for 40 thousand generally known English word lemmas. *Behavior Research Methods*, 46(3):904–911.

Cook, P. and Stevenson, S. (2010). Automatically identifying changes in the semantic orientation of words. In Calzolari, N., Choukri, K., Maegaard, B., Mariani, J., Odijk, J., Piperidis, S., Rosner, M., and Tapias, D., editors, *Proceedings of the Seventh International Conference on Language Resources and Evaluation (LREC'10)*, Valletta, Malta. European Language Resources Association (ELRA).

Fellbaum, C. (2005). Wordnet and wordnets. In Brown, K. et al., editors, *Encyclopedia of Language and Linguistics*, pages 665–670. Elsevier, Oxford, second edition.

Haslam, N. (2016). Concept creep: Psychology's expanding concepts of harm and pathology. *Psychological Inquiry*, 27(1):1–17.

Haslam, N., Dakin, B. C., Fabiano, F., McGrath, M. J., Rhee, J., Vylomova, E., Weaving, M., and Wheeler, M. A. (2020). Harm inflation: Making sense of concept creep. *European Review of Social Psychology*, 31(1):254–286.

Kay, C., Alexander, M., Dallachy, F., Roberts, J., Samuels, M., and Wotherspoon, I. (2020). The historical thesaurus of English (2nd edition, version 5.0). University of Glasgow. `https://ht.ac.uk`.

Warriner, A. B., Kuperman, V., and Brysbaert, M. (2013). Norms of valence, arousal, and dominance for 13,915 English lemmas. *Behavior Research Methods*, 45(4):1191–1207.

Xu, Y., Malt, B. C., and Srinivasan, M. (2017). Evolution of word meanings through metaphorical mapping: Systematicity over the past millennium. *Cognitive Psychology*, 96:41–53.
